# Supplementary material for: The pregnant myometrium is epigenetically activated at contractility-driving gene loci prior to the onset of labor in mice
Source: PLoS Biol. 2020 Jul 15;18(7):e3000710. doi: 10.1371/journal.pbio.3000710 (PMC7384763; doi:10.1371/journal.pbio.3000710)
Supplement: S1 Fig — Hierarchical clustering of RNA-seq samples from d15 and d19 when in active labor. Darker color indicates increased correlation. Data associated with this figure can be found in S1 Data. d, day; RNA-seq, RNA-sequencing. (PDF) [file pbio.3000710.s001.pdf]

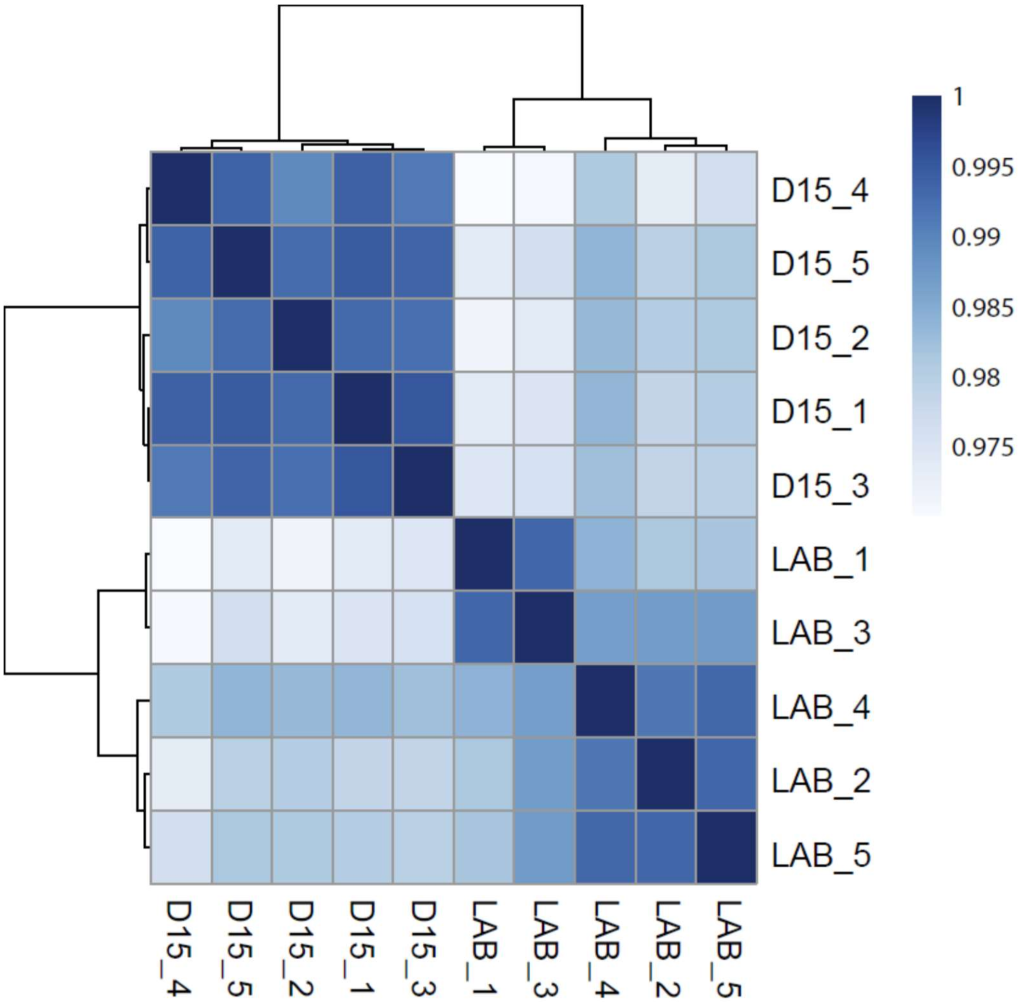

S1 Fig. Gestational timepoint specific RNA-seq samples cluster based on gestational timepoint of sample collection. Hierarchical clustering of RNA-seq samples from d15 and d19 when in active labour. Darker colour indicates increased correlation. Data associated with this figure can be found in S1 DATA.
