# Supplementary material for: The pregnant myometrium is epigenetically activated at contractility-driving gene loci prior to the onset of labor in mice
Source: PLoS Biol. 2020 Jul 15;18(7):e3000710. doi: 10.1371/journal.pbio.3000710 (PMC7384763; doi:10.1371/journal.pbio.3000710)
Supplement: S2 Fig — Applied anti-H3K27ac ChIP in murine myometrial tissue (target, pink) and murine embryonic stem cells (cell control, blue) revealed enrichment or lack of enrichment at select gene targets, as expected. Cell-specific enrichment of this histone mark observed at gene promoters expected to be active predominantly in myometrium rather than embryonic stem cells (left), in both cell types (center), and in neither cell type (right). Data associated with this figure can be found in S4 Data. ChIP, chromatin immunoprecipitation; H3K27ac, H3 acetylation on lysine residue 27. (PDF) [file pbio.3000710.s002.pdf]

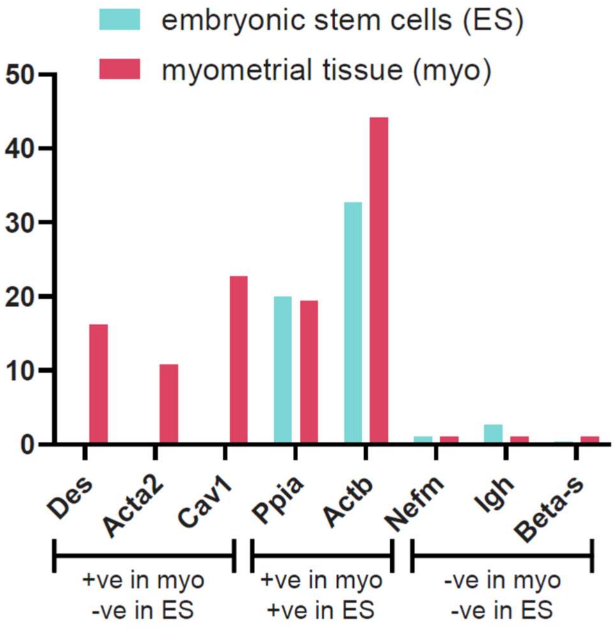

S2 Fig. Proof of ChIP selectivity in myometrial tissues. Applied anti-H3K27ac ChIP in murine myometrial tissue (target, pink) and murine embryonic stem cells (cell control, blue) revealed enrichment or lack of enrichment at select gene targets, as expected. Cell-specific enrichment of this histone mark observed at gene promoters expected to be active predominantly in myometrium rather than embryonic stem cells (left), in both cell types (center) and in neither cell type (right). Data associated with this figure can be found in S4 DATA.
