# Supplementary material for: The pregnant myometrium is epigenetically activated at contractility-driving gene loci prior to the onset of labor in mice
Source: PLoS Biol. 2020 Jul 15;18(7):e3000710. doi: 10.1371/journal.pbio.3000710 (PMC7384763; doi:10.1371/journal.pbio.3000710)
Supplement: S3 Fig — Metagene plots displaying H3K4me3 or H3K27ac enrichment +/− 2 kb of TSSs for genes in expression quartiles reveals increased modification at the promoters of highly expressed genes. Data associated with this figure can be found in S5 Data. H3K4me3, H3 trimethylation of lysine residue 4; H3K27ac, H3 acetylation on lysine residue 27; TSSs, transcription start site. (PDF) [file pbio.3000710.s003.pdf]

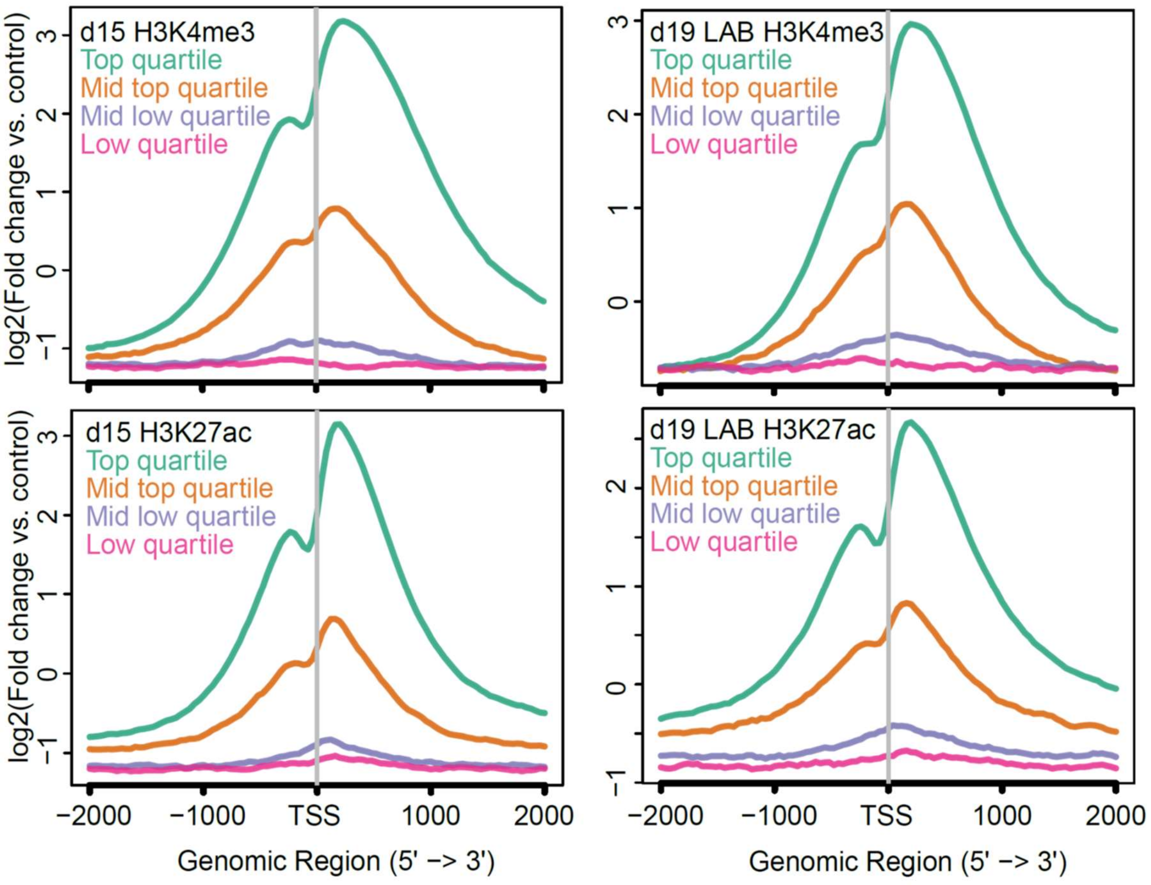

S3 Fig. Enrichment of activating histone marks at gene promoters depends on transcriptional status of genes. Metagene plots displaying H3K4me3 or H3K27ac enrichment +/- 2kb of TSS for genes in expression quartiles reveals increased modification at the promoters of highly expressed genes. Data associated with this figure can be found in S5 DATA.
