# Supplementary material for: The pregnant myometrium is epigenetically activated at contractility-driving gene loci prior to the onset of labor in mice
Source: PLoS Biol. 2020 Jul 15;18(7):e3000710. doi: 10.1371/journal.pbio.3000710 (PMC7384763; doi:10.1371/journal.pbio.3000710)
Supplement: S4 Fig — UCSC genome browser views of epigenetic and transcription regulatory mark enrichment profiles at (A) Fosl2, (B) Gja1, (C) Oxtr, and (D) Ptgs2. Data displayed include H3K4me3 signal (green), H3K27ac signal (blue), and RNAPII signal (burgundy) at d15, TNIL, LAB, and pp stages. Reads are mapped to mm10 assembly. d, day; Fosl2, Fos-like antigen 2; Gja1, Gap junction alpha 1; H3K4me3, H3 trimethylation of lysine residue 4; H3K27ac, H3 acetylation on lysine residue 27; LAB, active labor; pp, postpartum; Oxtr, Oxytocin receptor; Ptgs2, Prostaglandin G/H Synthase 2; RNAPII, RNA polymerase II; TNIL, term-not-in-labor; UCSC, University of California Santa Cruz. (PDF) [file pbio.3000710.s004.pdf]

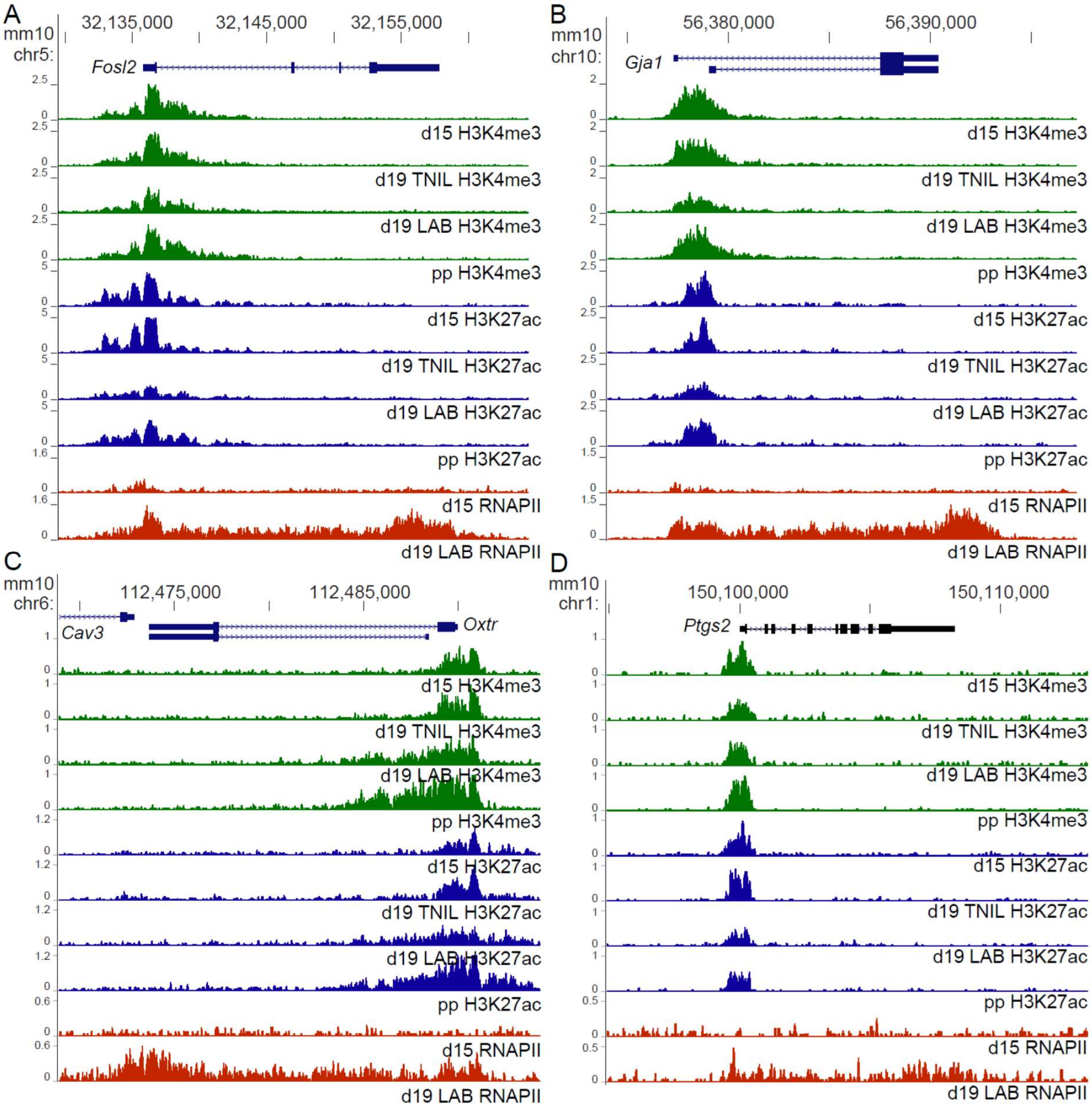

S4 Fig: Epigenetic landscapes of select labor-associated genes. UCSC genome browser views of epigenetic and transcription regulatory mark enrichment profiles at (A) Fosl2, (B) Gja1, (C) Oxtr, and (D) Ptgs2. Data displayed includes H3K4me3 signal (green), H3K27ac signal (blue), and RNAPII signal (burgundy) at d15, TNIL, LAB, and pp stages. Reads are mapped to mm10 assembly.
