# Supplementary material for: The pregnant myometrium is epigenetically activated at contractility-driving gene loci prior to the onset of labor in mice
Source: PLoS Biol. 2020 Jul 15;18(7):e3000710. doi: 10.1371/journal.pbio.3000710 (PMC7384763; doi:10.1371/journal.pbio.3000710)
Supplement: S5 Fig — Heatmaps representing promoters clustered according to H3K27ac and H3K4me3 enrichment levels, as per K-means clustering analysis. d15, TNIL, LAB, and pp time points indicated accordingly. H3K4me3, H3 trimethylation of lysine residue 4; H3K27ac, H3 acetylation on lysine residue 27; LAB, active labor; pp, postpartum; TNIL, term-not-in-labor. (PDF) [file pbio.3000710.s005.pdf]

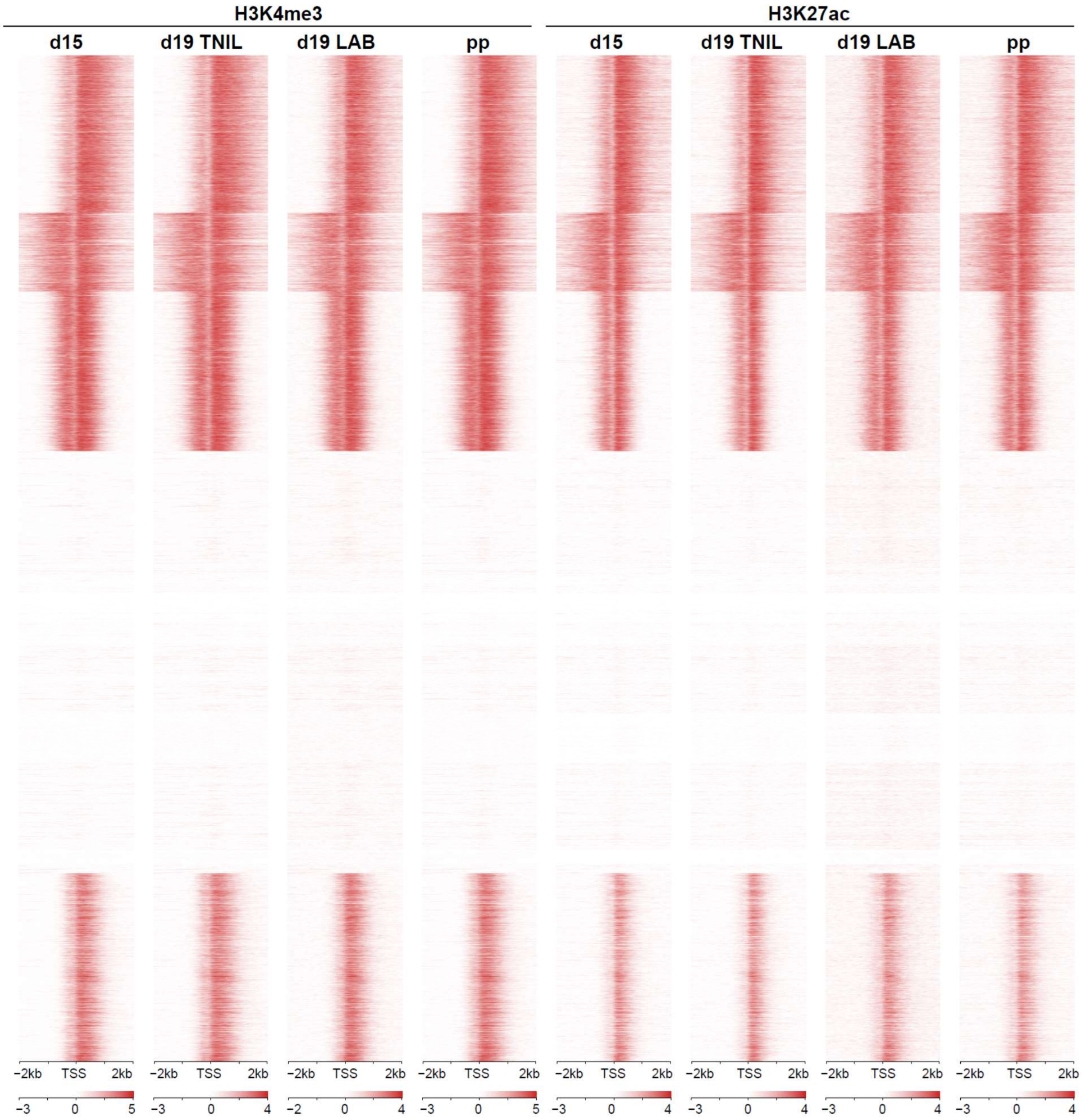

S5 Fig: H3K27ac and H3K4me1 enrichment levels at promoters in the myometrial genome exhibit no gestational age-specific enrichment trends. Heatmaps representing promoters clustered according to H3K27ac and H3K4me3 enrichment levels, as per K-means clustering analysis. D15, TNIL, LAB, and pp timepoints indicated accordingly.
