# Supplementary material for: The pregnant myometrium is epigenetically activated at contractility-driving gene loci prior to the onset of labor in mice
Source: PLoS Biol. 2020 Jul 15;18(7):e3000710. doi: 10.1371/journal.pbio.3000710 (PMC7384763; doi:10.1371/journal.pbio.3000710)
Supplement: S7 Fig — Metagene plots displaying RNAPII enrichment at genes in expression quartiles reveals increased association at the promoters and gene bodies of highly expressed genes. Data associated with this figure can be found in S5 Data. RNAPII, RNA polymerase II. (PDF) [file pbio.3000710.s007.pdf]

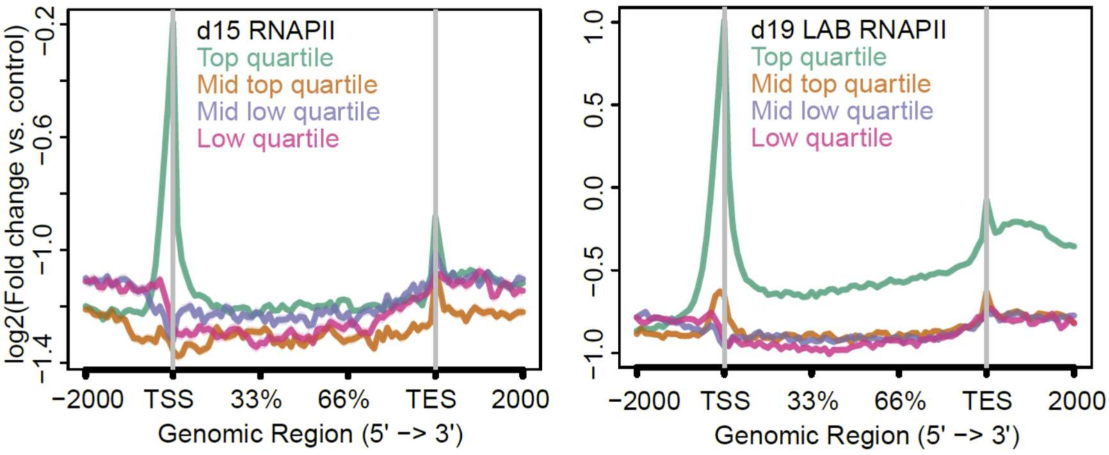

S7 Fig. Enrichment of RNAPII at gene bodies depends on transcriptional status of genes. Metagene plots displaying RNAPII enrichment at genes in expression quartiles reveals increased association at the promoters and gene bodies of highly expressed genes. Data associated with this figure can be found in S5 DATA.
