# Supplementary material for: The pregnant myometrium is epigenetically activated at contractility-driving gene loci prior to the onset of labor in mice
Source: PLoS Biol. 2020 Jul 15;18(7):e3000710. doi: 10.1371/journal.pbio.3000710 (PMC7384763; doi:10.1371/journal.pbio.3000710)
Supplement: S8 Fig — Motif enrichment at promoters (1 kb upstream of TSSs) of genes with increased expression in labor based on intron reads. Data associated with this figure can be found in S6 Data. TSSs, transcription start site. (PDF) [file pbio.3000710.s008.pdf]

Rank	Motif	Name	P-value	log P-pvalue	q-value (Benjamini)	# Target Sequences with Motif	% of Targets Sequences with Motif	# Background Sequences with Motif	% of Background Sequences with Motif
1	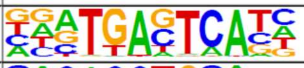	Fra1(bZIP)/BT549-Fra1-ChIP-Seq(GSE46166)/Homer	1e-4	-1.126e+01	0.0056	103.0	26.01%	5183.0	17.38%
2	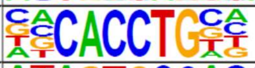	E2A(bHLH),near_PU.1/Bcell-PU.1-ChIP-Seq(GSE21512)/Homer	1e-4	-1.077e+01	0.0056	228.0	57.58%	14044.6	47.10%
3	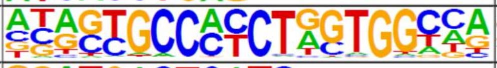	CTCF(Zf)/CD4+-CTCF-ChIP-Seq(Barski_et_al.)/Homer	1e-4	-1.039e+01	0.0056	41.0	10.35%	1548.6	5.19%
4	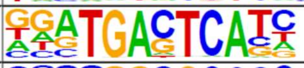	Fra2(bZIP)/Striatum-Fra2-ChIP-Seq(GSE43429)/Homer	1e-4	-9.835e+00	0.0059	89.0	22.47%	4462.7	14.97%
5	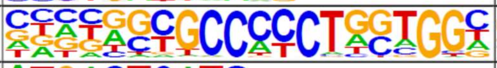	BORIS(Zf)/K562-CTCFL-ChIP-Seq(GSE32465)/Homer	1e-4	-9.772e+00	0.0059	58.0	14.65%	2561.2	8.59%
6	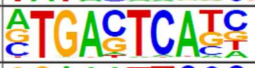	AP-1(bZIP)/ThioMac-PU.1-ChIP-Seq(GSE21512)/Homer	1e-4	-9.317e+00	0.0066	126.0	31.82%	6976.5	23.40%
7	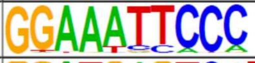	NFkB-p65-Rel(RHD)/ThioMac-LPS-Expression(GSE23622)/Homer	1e-3	-8.971e+00	0.0080	23.0	5.81%	713.8	2.39%
8	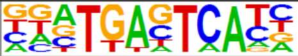	Fos(bZIP)/TSC-Fos-ChIP-Seq(GSE110950)/Homer	1e-3	-8.007e+00	0.0183	100.0	25.25%	5430.3	18.21%
9	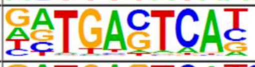	JunB(bZIP)/DendriticCells-Junb-ChIP-Seq(GSE36099)/Homer	1e-3	-7.648e+00	0.0233	95.0	23.99%	5153.5	17.28%
10	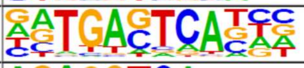	Fosl2(bZIP)/3T3L1-Fosl2-ChIP-Seq(GSE56872)/Homer	1e-3	-7.546e+00	0.0233	63.0	15.91%	3104.2	10.41%
11	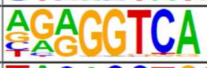	COUP-TFII(NR)/Artia-Nr2f2-ChIP-Seq(GSE46497)/Homer	1e-3	-7.445e+00	0.0234	242.0	61.11%	15751.3	52.83%
12	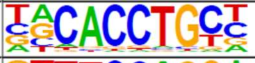	Snail1(Zf)/LS174T-SNAIL1.HA-ChIP-Seq(GSE127183)/Homer	1e-3	-7.320e+00	0.0243	153.0	38.64%	9204.1	30.87%
13	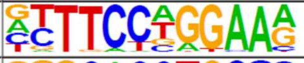	STAT4(Stat)/CD4-Stat4-ChIP-Seq(GSE22104)/Homer	1e-3	-7.248e+00	0.0243	172.0	43.43%	10581.6	35.49%
14	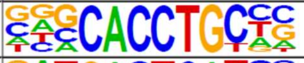	Slug(Zf)/Mesoderm-Snai2-ChIP-Seq(GSE61475)/Homer	1e-3	-7.120e+00	0.0254	108.0	27.27%	6111.7	20.50%
15	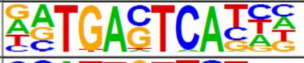	Atf3(bZIP)/GBM-ATF3-ChIP-Seq(GSE33912)/Homer	1e-2	-6.872e+00	0.0304	107.0	27.02%	6086.9	20.41%
16	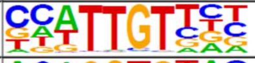	Sox6(HMG)/Myotubes-Sox6-ChIP-Seq(GSE32627)/Homer	1e-2	-5.901e+00	0.0752	225.0	56.82%	14811.9	49.67%
17	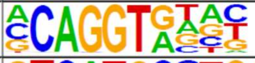	ZEB1(Zf)/PDAC-ZEB1-ChIP-Seq(GSE64557)/Homer	1e-2	-5.886e+00	0.0752	246.0	62.12%	16416.5	55.06%
18	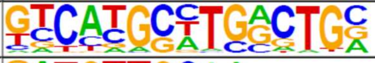	Pax8(Paired,Homeobox)/Thyroid-Pax8-ChIP-Seq(GSE26938)/Homer	1e-2	-5.518e+00	0.0982	64.0	16.16%	3446.7	11.56%
19	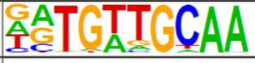	CEBP:AP1(bZIP)/ThioMac-CEBPb-ChIP-Seq(GSE21512)/Homer	1e-2	-5.352e+00	0.1097	121.0	30.56%	7351.9	24.66%
20	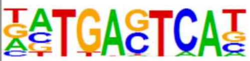	BATF(bZIP)/Th17-BATF-ChIP-Seq(GSE39756)/Homer	1e-2	-4.928e+00	0.1593	102.0	25.76%	6117.2	20.52%
21	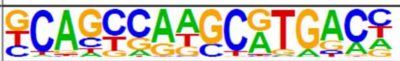	PAX5(Paired,Homeobox)/GM12878-PAX5-ChIP-Seq(GSE32465)/Homer	1e-2	-4.834e+00	0.1667	69.0	17.42%	3889.7	13.04%
22	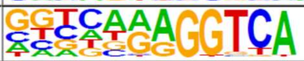	COUP-TFII(NR)/K562-NR2F1-ChIP-Seq(Encode)/Homer	1e-2	-4.832e+00	0.1667	212.0	53.54%	14108.7	47.32%
23	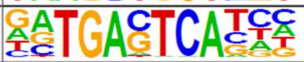	Jun-AP1(bZIP)/K562-cJun-ChIP-Seq(GSE31477)/Homer	1e-2	-4.724e+00	0.1699	42.0	10.61%	2151.0	7.21%
24	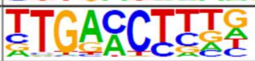	RARa(NR)/K562-RARa-ChIP-Seq(Encode)/Homer	1e-2	-4.722e+00	0.1699	316.0	79.80%	22230.8	74.56%

S8 Fig. Motif enrichment at promoters of labour upregulated genes. Motif enrichment at promoters (1 kb upstream of TSS) of genes with increased expression in labour based on intron reads. Data associated with this figure can be found in S6 DATA.
