# Supplementary material for: The pregnant myometrium is epigenetically activated at contractility-driving gene loci prior to the onset of labor in mice
Source: PLoS Biol. 2020 Jul 15;18(7):e3000710. doi: 10.1371/journal.pbio.3000710 (PMC7384763; doi:10.1371/journal.pbio.3000710)
Supplement: S5 Table — ChIP, chromatin immunoprecipitation; H3K4me3, H3 trimethylation of lysine residue 4; H3K27ac, H3 acetylation on lysine residue 27. (PDF) [file pbio.3000710.s023.pdf]

S5 Table. Correlation of gestational timepoint replicates in H3K4me3 and H3K27ac targeted ChIP samples.

sample	K4me3_D15_A	K4me3_D15_B	K4me3_TNIL_A	K4me3_TNIL_B	K4me3_LAB_A	K4me3_LAB_B	K4me3_1PP_A	K4me3_1PP_B
**K4me3_D15_A**	1	0.97	0.89	0.96	0.89	0.89	0.93	0.92
**K4me3_D15_B**	0.97	1	0.9	0.97	0.9	0.91	0.94	0.94
**K4me3_TNIL_A**	0.89	0.9	1	0.91	0.9	0.85	0.9	0.85
**K4me3_TNIL_B**	0.96	0.97	0.91	1	0.91	0.91	0.94	0.94
**K4me3_LAB_A**	0.89	0.9	0.9	0.91	1	0.89	0.92	0.87
**K4me3_LAB_B**	0.89	0.91	0.85	0.91	0.89	1	0.89	0.93
**K4me3_1PP_A**	0.93	0.94	0.9	0.94	0.92	0.89	1	0.91
**K4me3_1PP_B**	0.92	0.94	0.85	0.94	0.87	0.93	0.91	1
**sample**	**K27ac_D15_A**	**K27ac_D15_B**	**K27ac_TNIL_A**	**K27ac_TNIL_B**	**K27ac_LAB_A**	**K27ac_LAB_B**	**K27ac_1PP_A**	**K27ac_1PP_B**
**K27ac_D15_A**	1	0.86	0.83	0.85	0.83	0.8	0.79	0.73
**K27ac_D15_B**	0.86	1	0.74	0.88	0.79	0.81	0.81	0.82
**K27ac_TNIL_A**	0.83	0.74	1	0.79	0.76	0.8	0.75	0.71
**K27ac_TNIL_B**	0.85	0.88	0.79	1	0.81	0.82	0.83	0.8
**K27ac_LAB_A**	0.83	0.79	0.76	0.81	1	0.85	0.87	0.79
**K27ac_LAB_B**	0.8	0.81	0.8	0.82	0.85	1	0.85	0.82
**K27ac_1PP_A**	0.79	0.81	0.75	0.83	0.87	0.85	1	0.88
**K27ac_1PP_B**	0.73	0.82	0.71	0.8	0.79	0.82	0.88	1
