# Supplementary material for: The pregnant myometrium is epigenetically activated at contractility-driving gene loci prior to the onset of labor in mice
Source: PLoS Biol. 2020 Jul 15;18(7):e3000710. doi: 10.1371/journal.pbio.3000710 (PMC7384763; doi:10.1371/journal.pbio.3000710)
Supplement: S13 Table — ChIP-qPCR, ChIP-quantitative polymerase chain reaction. (PDF) [file pbio.3000710.s031.pdf]

S13 Table. List of primers used in ChIP-qPCR test.

Gene Targets	Target Region	Forward Sequence (5'->3')	Reverse Sequence (5'->3')	Amplicon Size (bp)
Desmin	gene body	GACGCTGTGAACCAGGAGTT	GTAGTTGGCGAAGCGGTCAT	84
Acta2	gene promoter	ACACATTTCCAGCATAGGACACC	AGGTAGTTGCCTGCTCTCTGATG	94
Ppia	gene promoter	TGTTCGAGTTTCCGCAGAGAG	TTGCACAGAGCAAGTAACTGAGG	105
Caveolin-1	gene body	TGAAAAGCTAGGAATGTCTTAGGG	CGAACGTGTCATCTGGAAAACT	117
Actb	gene promoter	CTAGGCGTAAAGTTGGCTGTG	CTCTCGTGGCTAGTACCTCACTG	120
Nefm	gene exon	CAGCACCGTGTCCTCCTCCT	GGCTGAAGTCGAGGCTGCTC	101
Beta-s	gene exon	TTCTGACAGACTCAGGAAGAAACCA	AGCACCATGGCCACCAATCT	93
