# Supplementary material for: The pregnant myometrium is epigenetically activated at contractility-driving gene loci prior to the onset of labor in mice
Source: PLoS Biol. 2020 Jul 15;18(7):e3000710. doi: 10.1371/journal.pbio.3000710 (PMC7384763; doi:10.1371/journal.pbio.3000710)
Supplement: S14 Table — RT-qPCR, reverse transcriptase-quantitative polymerase chain reaction. (PDF) [file pbio.3000710.s032.pdf]

S14 Table. List of primers used in RT-qPCR experiments.

Gene Target	Target Region	Forward Sequence (5'->3')	Reverse Sequence (5'->3')	Amplicon Size (bp)
Fosl2	exon-intron boundary	TATCCACGCTCACATCCCTACA	TGTCCCCCAGCTACCAACATA	177
Fos	exon-intron boundary	TGGAGGTGACACTAGACAACCTT	AGTGTATCTGTCAGCTCCCTCCT	153
Gja1	exon-intron boundary	TGAAACCATCAATTTACAGTCTACAA	GTTCATCACCCCAAGCTGACT	226
Oxtr	exon-intron boundary	GGGAGTCCAGAGATAGTGGAAGTA	TTATCTCCAAGGCCAAAATCC	194
Ptgs2	exon-intron boundary	TTGAAGACCAGGAGTACAGCTTC	CAAAAATCCTAAAGCTACTGACCA	166
Hist1	within exon	GGCCAAGGCTTCCAAGAAGT	CCACCTTGTAGTGGCTCTTGATA	137
