# Supplementary material for: The pregnant myometrium is epigenetically activated at contractility-driving gene loci prior to the onset of labor in mice
Source: PLoS Biol. 2020 Jul 15;18(7):e3000710. doi: 10.1371/journal.pbio.3000710 (PMC7384763; doi:10.1371/journal.pbio.3000710)
Supplement: Attachment — Submitted filename: Shchuka and Abatti 2020 Response to Reviewers_May19.docx [file pbio.3000710.s033.docx]

Response to Reviewer #1:

**Reviewer #1:** The manuscript by Shchuka and colleagues assessed whether or not the pregnant mouse myometrium was epigenetically activated at contractility-driving gene loci just prior to labour. This appears to be a revised manuscript so it is not known what was revised since a marked version was not provided, but it was very well presented. The work is excellently written with a solid introduction, excellent rationale and objective. The figures were well laid out, well described and appropriately interpreted. Overall, the manuscript significantly advances our understanding of the epigenetic and transcriptional alterations that occur in uterine smooth muscle prior to parturition. I only have one minor comment to provide.

**RESPONSE: We would like to thank the reviewer for their overall favorable assessment of our work.**

Minor Comment
1.      In the Discussion, lines 334-336, the authors indicated that the results explain the effortlessness with which labour an occur before term. The authors should take the opportunity to speculate a little bit as to why that would be an evolutionary advantage or advantage otherwise. Would this allow the pregnant dam to abort their fetus[es] in the event of a stressful, dangerous or other situation since the DNA is open and accessible and just needs a specific event[s] to set in motion labour? Could this be a survival strategy for the dam in a way?

**RESPONSE: As was suggested, we have expanded upon our discussion section to consider our results pertaining to DNA accessibility during pregnancy in the context of evolutionary perspectives surrounding preterm birth in the current scholarly literature. We give consideration to the theories that preterm birth evolved as an adaptive response to optimize maternal and/or fetal survival, while also acknowledging the alternative position of viewing preterm birth as a pathophysiological condition. Because our results do not support one theory over another, our revised manuscript does not take a side, but instead acknowledges both perspectives.**

**The manuscript now states [lines 390-409], “****Though presenting an inaugural view of the myometrial epigenetic landscape, the already validated pronounced enrichment of two prominent activation-associated histone marks at labor-associated gene loci as early as day 15 does itself suggest the following: that such a molecular set-up may explain the ease with which labor can occur in advance of term, if portions of the genome that are critical for contractility onset already contain accessible chromatin. This notion presents interesting implications for opposing critical conceptions of preterm birth, which characterize the event either as a pathological deviant version of its healthy default norm or as an evolutionary adaptive response that can act as a survival mechanism for the mother and/or fetus [PMID: 30966892]. From the former perspective, epigenetic activation of chromatin in myometrial cells at the start of the late gestation period allows for early locus access to transcriptional machinery, but a prematurely-timed stimulus prior to the end of term detrimentally activates the contraction-associated expression program too early, resulting in delivery of the fetus before term. As for the latter viewpoint, early access to genomic regions within labor-associated gene loci allows the dam to give birth earlier if such timing provides some kind of fitness advantage. For instance, the fetus’ exit from unfavourable *in utero* conditions may be enabled via preterm delivery to promote its survival, at the risk of subjecting the underdeveloped pup to potentially intolerable *ex utero* conditions. Alternatively, some have argued that the dam may induce an early birth as a pregnancy termination strategy if fetal complications are not deemed worth the cost of the especial maternal energy investment in the late gestational period [PMID: 30966892]. In either speculative case, the epigenetic priming event we have observed offers a novel mechanistic angle from which to view the potential initiation of preterm birth.”**

Response to Reviewer #2:

**Reviewer #2:**    In this manuscript, the authors performed simultaneous RNA-seq and ChIP-seq analysis of pregnant [days [d]15, d19-not-in-labor [NIL], d19-in labor [LAB]] and post-partum C57Bl/6 mouse myometrium to assess changes in gene expression in association with genome-wide binding of the active histone marks, H3K27ac and H3K4me3, and of RNA-polymerase II [RNAPII, S5-p] to promoters and distal enhancers. They identified 533 genes [including known contraction-associated genes] that were upregulated at term, compared to d15. Surprisingly, the promoters and potential intergenic enhancers of these genes manifested enrichment of the active H3K27ac and H3K4me3 histone marks as early as d15, and binding of these histone marks remained relatively unchanged at d19-NIL and d19-LAB. By contrast, binding of RNAPII to the promoters and potential distal enhancers of contraction-associated genes was markedly increased at d19-LAB, compared to d15. Based on these findings, the authors
conclude that 'epigenetic activation of the myometrial genome precedes active labor by at least 4 days in the mouse, suggesting that these genes are poised for rapid transcriptional activation at term.' Since the promoter and enhancer regions are enriched in AP-1 motifs, and since Fos is one of the labor-associated genes in myometrium, an elaborate model is presented, indicating Jun binding in both quiescent and laboring myometrium, and enhanced Fos and RNAPII recruitment to both promoters and distal enhancers during labor.

        The studies are well executed and the manuscript is clearly written; however, the epigenetic mechanisms that lead to the increase in RNAPII binding and transcriptional activation of the labor-associated genes at term remain undefined. While an interesting hypothesis is proposed, a number of essential components are lacking from this study to support this. These include the following:

Major points:

1.       The assumption that chromatin is 'open' in myometrium as early as d15 is based on the finding that binding of the active histone marks, H3K27ac and H3K4me3 don't change between d15 and d19-LAB. However, to what is d15 being compared? It would be important to compare binding of these histone marks to an earlier gestational time point [i.e. is binding at d15 significantly increased compared to d5 or d10?], and to non-pregnant myometrium. Rather than repeating the ChIP-seq study, the authors could select promoter regions of several known contraction-associated genes and use ChIP-qPCR to analyze binding of these modified histones.

**RESPONSE: We agree that whether or not labour-associated gene promoters contain an enriched H3K27ac- and H3K4me3-marked epigenetic signature even earlier than d15, and at what point such marks are acquired, are important questions raised by our results. Comparing the profiles for both marks in the gestational age-specific data we have generated to the profiles in earlier time points could address these questions; however, we do not currently have, nor can we currently generate, data for said earlier timepoints due to COVID19-related lab closure. Instead, we have discussed these points, as per associate editor Dr. Jiang’s recommendation, in the discussion section of the revised manuscript version. Briefly, we acknowledge and summarize the reasons underlying the significance of investigations into H3K27ac and H3K4me3 mark enrichment at labor-associated gene promoters at earlier timepoints, with non-pregnant myometrial samples as a control to better inform our gestational stage-related observations.**

**The manuscript now states [lines 357-371], “The precise timepoint at which labor-driving regulatory regions begin to exhibit the activating histone profile we observed, however, remains to be determined. Though taking place in the final quarter of the murine gestational period, the gestational day 15 timepoint nevertheless corresponds to a myometrial cellular state of dormancy. This non-contractile phenotype is accompanied by a substantially disparate transcriptome from its contractile counterpart, as both our work here and prior studies [PMIDs: 27291689, 26485220, 24273302] have demonstrated****. In light of these molecular-level expression and tissue-level phenotypic differences, we initially hypothesized that the promoters of labor-upregulated genes would not have acquired activating histone marks on day 15. Our finding to the contrary raises the possibility that labor-associated regions are enriched by H3K27ac and H3K4me3 modifications still earlier than this day. Whether these activating marks are first acquired at a timepoint during early or mid-gestation, or, perhaps more intriguingly, whether such epigenetic profiles exist in the non-pregnant state and persist throughout gestation, requires further investigation. Comparing the profiles of labor-driving gene loci in pregnant mouse myometrium at gestational timepoints prior to day 15 with those of myometrium in non-pregnant female mice would definitively ascertain what window of time, if any, precedes the establishment of activating histone mark deposition at critical regulatory elements.”**

2.      No evidence is presented that chromatin is actually 'open' at d15. Certainly, the two histone marks chosen [H3K27ac and H3K4me3] may not tell the whole story. Binding of the active histone marks H3K9ac, H3K14ac, as well as the repressive marks, H3K9me2/3 and H3K27me3, should be analyzed, as further indices of active and inactive chromatin. This again could be performed using ChIP-qPCR of selected contraction-associated genes at a range of gestational time points. Binding of endogenous selected histone deacetylases [HDACs] also may provide important insight regarding the regulation of dynamic changes in chromatin accessibility. Additional time points between d15 and d19-LAB should be analyzed, since one would expect activating epigenetic changes to occur prior to increased RNAPII binding and increased gene transcription

**RESPONSE: We agree with the reviewer’s statements that we need to be careful about labelling the chromatin as “open” on the basis of only two histone marks’ enrichment patterns, and that examination of additional histone marks at these and other late gestational timepoints would allow for a more comprehensive view of the time-specific extent of “openness” of chromatin at our regions of interest in the myometrium. To address this issue, we have expanded our discussion to acknowledge the need for further inquiry into the active and repressive histone binding profile at labor-associated genes in future studies, with a particular emphasis on acquiring the timepoints at which contractility-driving gene promoters might exhibit a bivalent histone signature that poises said promoters and enhancers for activation [PMIDs: 16630819; 26553936].**

**The manuscript now states [lines 372-389], “Our study’s H3K27ac and H3K4me3 enrichment data at both promoters and intergenic regions provide a critical first look at the state of chromatin in myometrial cells at different gestational stages. Future consideration of other epigenetic markers can more discriminately trace significant chromatin-altering events that occur during pregnancy. For instance, apart from H3K4me3, gene promoters undergoing a readying process for strong expression induction are characterized by pronounced enrichment of additional modifications, including acetylation of lysine residues 9 and 14 on histone H3 (active markers H3K9ac and H3K14ac)** **[PMIDs: 16630819; 26553936]. Furthermore, “bivalent promoters,” or promoters simultaneously laden with H3K4me3 and tri-methylation of lysine residue 27 on H3 (repressive marker H3K27me3), have been shown to designate genes poised for activation [PMIDs: 26553936****; 22920947; 31818514].** **In the case of intergenic regions, the H3K27ac marker is characteristically thought to distinguish active enhancers from their inactive or poised counterparts [PMID: 21106759].** **Our experimental results imply that this classic model does not faithfully apply in select physiological circumstances. Uncovering the timepoint-specific enrichment of inactive or poised enhancer-associated histone markers, such as histone H3 mono-methylation of lysine residue 4 (H3K4me1) and H3K27me3 [PMIDs: 21106759; 21632746], would pinpoint more precisely which intergenic regions display an enhancer signature and when such regions become active. Supplemented with our current established H3K27ac and H3K4me3 enrichment profiles, a further tracing of promoters’ and enhancers’ gain, maintenance, or loss of other histone marks in a time-course-dependent manner can offer a more developed view of the progressive changes in chromatin state at key contractility-driving genes.”**

**We have also reviewed the manuscript and removed any statements that declaratively affirm or strongly suggest that our experiments have established chromatin is open at any point in time. We have, however, maintained that the presence of H3K27ac and H3K4me3 at critical genomic regions that we have observed as early as day 15 may increase accessibility to these regions, a claim that is in line with what we know about the activating propensity of these histone modifications and that remains reasonable even if one or more of the genomic regions in question are at any of the gestational timepoints we tested simultaneously enriched by repressive histone marks.**

3.      The hypothesis set forth in the text and in Figure 5 suggests that Jun is bound during myometrial quiescence and that increased Fos expression and promoter/enhancer binding increases near term, resulting in enhanced RNAPII binding and transcriptional activation of contractile genes. However, this is conjecture. Temporal changes in the binding of endogenous Jun and Fos and their association with active/inactive histone marks and RNAPII binding should be included to provide critical insight into the mechanisms underlying transcriptional activation of labor-associated genes in the pregnant myometrium.

**RESPONSE: We agree that portions of the model in Figure 5 are conjectural, and have modified the figure, its corresponding legend, and the first paragraph of the manuscript discussion to more clearly delineate which parts of our proposed model are supported by our data or the data of previous studies, and which parts are speculative. We agree that the gestational stage-specific JUN:JUN and FOS:JUN binding events we propose are not validated by the data we present in this paper, therefore we have indicated that such binding events are speculative in the text and by using designator question marks in the figure [modified Fig 5]. We also agree that, in the absence of transcription factor binding data, we have not experimentally validated that the proposed binding events stimulate RNAPII binding at contractility-driving gene loci. We have modified the text to more clearly outline that this putative proximal cause is also conjectural.**

**The manuscript now states, for instance:**

**[Lines 293-295]: “During pregnancy, promoters and intergenic regions may be bound by homodimerized members of the JUN transcription factor family, proteins that are already significantly expressed at this stage.”**

**[Lines 300-303]: “We suggest that these motifs, if resident in regions of open chromatin and formerly bound by JUN proteins, may allow for a laboring stage-specific replacement of homodimers with FOS:JUN family member heterodimers, as well as phosphorylated RELA [both of which have been implicated in labor onset].”**

**[Lines 313-316]: “The aforementioned regions also contain an enrichment of AP-1 transcription factor motifs that may allow for potential binding of homodimerized JUN proteins, which are already known to be expressed in the quiescent stage.”**

**[Lines 317-321]: “Putative regulatory region-bound JUN proteins may be replaced by FOS:JUN proteins alongside phosphorylated RELA. This hypothetical switch in dimerized TF binding events may underlie the labor-specific recruitment of RNAPII to labor-associated gene promoters that we have observed, and consequently enhance labor-specific transcription through the bodies of these genes.”**

Additional comments:
4.      Lines 165-167 and Figure 2D - This reviewer finds it very surprising that expression of the contraction associated genes were only significantly increased in laboring myometrium [d19-LAB], compared to d15. According to published studies, they should be increased by d19-NIL, but this does not appear to be the case.

**RESPONSE: In the existing scholarly literature, some discrepancies already exist between different studies with respect to the time-specific expression profiles of mRNA for select contraction-associated genes. Though we have examined the transcriptional status of the genes *Fos*, *Fosl2*, *Ptgs2*, *Gja1*, and *Oxtr* by monitoring primary transcript levels at different timepoints [one of which, *Fos*, does significantly exhibit an increased expression output by term-not-in-labour compared to day 15 in our results], our data in Figure 2D generally mirrors corresponding mRNA and/or protein expression data in previously published studies from more than one research group. These data include the following:**

**1] Nadeem et al. observed a statistically significant difference between FOSL2 protein levels in mouse myometrial nuclear fractions for term labour compared to term-not-in-labour or day 15 via Western blot [PMID:** **28945005].**

**2] MacIntyre et al. observed a statistically significant difference between CX43 [a.k.a. GJA1] protein levels in mouse myometrium at Day E18 and those in term labour via Western blot [PMID: 24497579].**

**3] Ou et al. observed a statistically significant difference between OTR [a.k.a. OXTR] levels in rat myometrium at gestational day 22 [the rat equivalent of term-not-in-labour] and at term labour, via RT-PCR [PMID: 9780309].**

**4] Although MacIntyre et al. observed increased levels of COX-2 [a.k.a. PTGS2] protein by Day E16, which persist throughout E18 and term via Western blot [PMID: 24497579], Migale et al. contrarily saw an ~5-fold increase in *Ptgs2* mRNA expression levels from E18 to term labour, compared to only a ~1.3 increase from E16 to E18 in their RNA-seq data [PMID: 27291689].**

5.      Figures 3 and 4 - The manuscript is highly focused on epigenetic changes within and surrounding the Fos gene. However, additional genome browser views of other differentially regulated contraction-associated genes should be included to compare gestational changes in binding of modified histones and RNAPII to support their conclusions.
**RESPONSE: We organized the experimental data in our manuscript to display the genome-wide histone, polymerase, and RNA expression trends across gestational stages, and then focused on *Fos* as our case study gene to demonstrate how the trends we have observed at a genome-wide level are reflected in a more localized context. We have maintained this organization in the revised version of our manuscript for the sake of clarity. However, we have included the requested additional genome browser views of four other genes – *Fosl2*, *Gja1*, *Oxtr*, and *Ptgs2* – in our supplemental data [now Supplemental Figure 4]. These other genes show similar trends to what was presented at the *Fos* gene locus.**

Response to Reviewer #3:

**Reviewer #3:** The manuscript "The pregnant myometrium is epigenetically activated at contractility-driving gene loci prior to the onset of labor in mice" by Shchuka et al investigates the chromatin states of the quiescent and contractile myometrial genome and correlates the changes with transcriptional events that result in the establishment of such states. Specifically, the authors identified a set of contractility-associated genes that are highly upregulated at active labor [E19] compared to pre-labour [E15] due to higher transcription. Using genome-wide approaches to explore histone modifications, the promoters associated with these genes show a very high level of active histone modifications such as H3K27ac and H3K4me3.  The main novel finding in this manuscript is that the level of H3K27ac was enriched at all time points [prelabour, labour and post-labor], suggesting that the transcription of labor-associated is not regulated by chromatin changes active histone marks [ie
the promoters are already in an active state]. The authors further show that the transcription of labor-associated genes correlated to high enrichment of RNPII, suggesting that although the promoters contain active histone marks, something else is recruiting RNAPII to drive transcription.  Thus, the mechanism driving transcription at labour is more complex. The authors also identified enhancers that have H3K27Ac in pre-labour and at labour, but also display increased enhancer RNA production and RNAPII only at labour. Using motif analysis of the promoters and enhancers, the authors suggest that AP-1 and RELA may play a role in driving transcription of genes at labour onset.
The manuscript is very well written and presents novel findings. The idea that labour genes have active promoters and enhancers and that an additional level of regulation controls transcriptional onset is extremely interesting. The quality of the data is high, including replicates as appropriate. However, I am less convinced that AP-1/RELA is the mechanism involved.  I have provided comments for the author outlined below.
Comments:
1.      The authors present the model that JUN family protein plays a role in maintaining myometrial gene expression during pregnancy but heterodimerization with FOS is required for regulation of labor associated genes. This model is very interesting. However, the authors fail to show this experimentally. If possible, data examining FOX/JUN and/or RELA binding would greatly strengthen the model. Motif analysis alone does not determine if binding is actually occurring. Thus, Figure 5, the model figure presented in the manuscript is not fully experimentally validated.

**RESPONSE: In response to this comment, we have modified Figure 5, its corresponding legend, and the first paragraph of the manuscript discussion to more clearly delineate which parts of our proposed model are supported by our data or the data of previous studies, and which parts are speculative. We agree that motif analyses do not in and of themselves confirm the binding of their corresponding TFs. FOS, JUN and/or RELA ChIP data are beyond the scope of this study, in our opinion, but are an important part of our research plans once our research lab reopens after the COVID-19 related shutdown. To clarify the parts of our model that are not yet validated, we have indicated that such binding events are speculative in the text and by using designator question marks in the figure [modified Fig 5].**

**The manuscript now states, for instance:**

**[Lines 293-295]: “During pregnancy, promoters and intergenic regions may be bound by homodimerized members of the JUN transcription factor family, proteins that are already significantly expressed at this stage.”**

**[Lines 300-303]: “We suggest that these motifs, if resident in regions of open chromatin and formerly bound by JUN proteins, may allow for a laboring stage-specific replacement of homodimers with FOS:JUN family member heterodimers, as well as phosphorylated RELA [both of which have been implicated in labor onset].”**

**[Lines 313-316]: “The aforementioned regions also contain an enrichment of AP-1 transcription factor motifs that may allow for potential binding of homodimerized JUN proteins, which are already known to be expressed in the quiescent stage.”**

**[Lines 317-321]: “Putative regulatory region-bound JUN proteins may be replaced by FOS:JUN proteins alongside phosphorylated RELA. This hypothetical switch in dimerized TF binding events may underlie the labor-specific recruitment of RNAPII to labor-associated gene promoters that we have observed, and consequently enhance labor-specific transcription through the bodies of these genes.”**

**However, prior studies have confirmed that JUN proteins homodimerize, FOS proteins heterodimerize with JUN proteins, and that these proteins, along with RELA, play a role in labour [PMIDs: 21734268; reviewed in 9069263], and we have therefore retained these elements in the model figure.**

2.      For the motif analysis, the authors identified motifs enriched in the promoters of labour onset genes. However, for this analysis they used random sequences for background. Would it be more appropriate to use all promoters or promoters of genes that are unchanged to show enrichment?

**RESPONSE: We apologize for the lack of clarity in the methods section of the text: our analysis did, in fact, involve the use of 1kb-length promoter sequences selected at random by the HOMER tool from a list of all promoter sequences that we provided as input, and *not* random genomic sequences of the same length, as our formerly submitted manuscript may have suggested. We have rephrased this part of our description in our revised manuscript’s motif enrichment analysis methodology section to more accurately reflect how the analysis was conducted.**

**The manuscript now states [lines 546-547], “Promoter sequences (-1kb of TSS) of upregulated genes at labor were compared against an input of randomly selected 1kb-length promoter sequences**.**”**

**We deem this input approach more appropriate for our analysis compared to the use of non-expression-changing gene promoters as input because the number of genes exhibiting similar expression levels between both timepoints is fairly small. Despite the fact that many genes do not pass our statistical cut-off for significant differential expression [4-fold change, P<0.01], a large fraction of these genes do exhibit comparative up- or down-regulated expression levels. The remaining number of genes with consistent expression levels across timepoints is too small for an adequate number of promoter sequences to be used as input.**

3.      Histone modification such as H3K4me1 has been used to identify enhancer and its overlap with H3K27ac determines the active state of the enhancer. Why did the authors not investigate the level of H3K4me1 to identify all enhancer and investigated their active, poised and inactive states at the different time points? This may help strengthen the enhancer part of the model by identifying enhancers in pre-labour. Please clarify.

**RESPONSE: Many studies have focused on locating putative enhancers in their tissue contexts of interest through an examination of intergenic regions co-enriched by both H3K27ac and H3K4me1 in particular cell types. In our work, we prioritized our focus on the genome-wide enrichment of H3K27ac because this marker’s associated peaks are typically correlated with specifically active enhancers [PMID: 21106759]. Furthermore, H3K27ac peaks have more recently been shown to be more predictive of active enhancers than H3K4me1 peaks [PMID: 30137428]. H3K4me1 in the absence of H3K27ac has been proposed to mark poised enhancers that later acquire H3K27ac. As we determined that at day 15 putative enhancers marked in labouring tissue by H3K27ac already have the H3K27ac mark the addition of H3K4me1 would not identify any poised enhancers that acquire H3K27ac at labour. We do acknowledge, however, that the collecting genome-wide data for enhancer-associated marks other than H3K27ac and eRNA would allow for a more precise discrimination of critical distal regulatory regions that may be involved in labor-associated gene regulation. Specifically, it would be advantageous to determine if H3K27ac marks are present in non-pregnant tissue at the same locations as in labouring tissue and, if not, whether these regions are poised with H3K4me1 marks.**

**The manuscript now states [lines 380-389], “In the case of intergenic regions, the H3K27ac marker is characteristically thought to distinguish active enhancers from their inactive or poised counterparts [PMID:** [**21106759**](https://www.ncbi.nlm.nih.gov/pubmed/21106759)**]. Our experimental results imply that this classic model does not faithfully apply in select physiological circumstances. Uncovering the timepoint-specific enrichment of inactive or poised enhancer-associated histone markers, such as histone H3 mono-methylation of lysine residue 4 (H3K4me1) and H3K27me3 [PMIDs:** [**21106759**](https://www.ncbi.nlm.nih.gov/pubmed/21106759)**;** [**21632746**](https://www.ncbi.nlm.nih.gov/pubmed/21632746)**], would pinpoint more precisely which intergenic regions display an enhancer signature and when such regions become active. Supplemented with our current established H3K27ac and H3K4me3 enrichment profiles, a further tracing of promoters’ and enhancers’ gain, maintenance, or loss of other histone marks in a time-course-dependent manner can offer a more developed view of the progressive changes in chromatin state at key contractility-driving genes.”**

4.      On page 11, the authors indicate that they are looking at "other intergenic regions of interest". That is too vague and should be clarified.

**RESPONSE: We have re-phrased the statement containing this phrase to specify that we mean that we have assessed non-coding regions at labour-associated gene loci that contain an epigenetic signature typical of enhancers.**

**The manuscript now states [lines 257-259], “Since intergenic regions containing RNAPII and H3K27ac peaks have been noted to occur at active enhancer regions [PMIDs: 17277777, 20485488, 19295514], we examined the loci of other labor-associated genes to see if they contained intergenic regions that similarly exhibit this epigenetic profile.”**

5.      It is not always clear for the histone mod analyses whether the authors are looking at all promoters, or only the promoters of the labour-onset genes. This should be clarified throughout the manuscript.

**RESPONSE: We have reviewed the manuscript and made the appropriate changes in any instances that did not clearly designate whether we were conducting genome-wide promoter analyses or labour-associated gene promoter analyses. In such textual instances, we have now more precisely indicated when we refer to all promoters across the genome [see changes in lines 182-185; 203-204; 262-265] or promoters of labour-upregulated genes [see changes in lines 222-224; 350-354].**

6.      Fig 1B, several genes are downregulated upon onset of labor. What are those genes and what are their cellular/molecular functions? The author could perform GO analysis of differentially expressed genes at these time points

**RESPONSE: We have performed a GO analysis for differentially up-regulated and down-regulated genes in our exon-based RNA expression datasets and have included the list of biological processes, molecular functions, cellular components, and key pathways in tabular format [now Supplemental Tables 2 and 3]. To prevent veering too far from the manuscript focus on the chromatin and epigenetic landscape, we have briefly referenced this GO search [and its myometrial-specific findings] in our main text.**

**The manuscript now states [lines 107-110], “Hierarchical clustering analysis of these genes confirmed similar expression trends from mice of the same gestational age (Fig 1C), while GO term analyses highlighted the involvement of down-regulated and up-regulated genes at term in myometrial relaxation and contraction pathways, respectively (S2 and 3 Tables).”**

7.      Figure 1B and 2B; add the number of genes in the figure.

**RESPONSE: We have added the numerical values representing the total number of down-regulated and up-regulated genes at the top corners of their corresponding panels in Figures 1B and 2B.**

8.      Figure 3B, why did the authors not perform K-means clustering to identify regions with different H3K27ac/H3K4me1 enrichments at d15, TNIL, d19 LAB, pp?

**RESPONSE:** **We did not initially perform the suggested method for our H3K27ac and H3K4me3 promoter binding data because the analyses we had conducted had already demonstrated that, across the genome, most promoter regions were not gaining or losing significant amounts of histone enrichment signal. Because of this lack of large-scale differences, we reasoned that K-means clustering would not be the most suitable method to present this finding, and instead presented our enrichment profile in Figure 3B in what we deemed a clearer way of demonstrating the overall similar histone trends across gestational timepoints. In our revised manuscript version, however, we have included the results of our K-means clustering analysis as a supplement [now Supplemental Fig 5] accompanying Figure 3B. These new figures again clearly show the overall similarity in H3K27ac and H3K4me3 enrichment at gene promoters across all four gestational timepoints.**

9.      Figure 4C; add H3K27ac, RNPII and eRNA signals as to the FOS genomic maps.

**RESPONSE: The signal tracks for H3K27ac and RNA have been added to the RNAPII data originally shown in Figure 4C [see modified Figure 4C]. The peaks called from these tracks are shown as well [notably, no peaks were called at this region in the d15 RNAPII data]. Note that because the eRNA levels are much lower than protein-coding gene expression levels in the locus, the genomic region containing the eRNA is shown on a different scale from the region spanning the *Fos* gene.**

10.     Consider adding motif analysis results in the main figures.

**RESPONSE: The motif analysis has been added to Figure 4 [see modified Figure 4E/F].**

11.     What are the mRNA levels of FOS/JUN/AP1 at different time-points? RNA-seq data can show their mRNA levels.

**RESPONSE: The relative mRNA levels of AP-1 factors at the laboring gestational timepoint compared to d15 are listed in Supplemental Tables 1 [exon-based reads] and 2 [intron-based reads]. We have also briefly alluded to the expression levels of several key AP-1 factors from our data in the modified discussion.**

**The manuscript now states [lines 414-416], “Indeed, our RNA-seq data accordingly show that levels of Jun and Jund mRNA are similar across both gestational timepoints, whereas transcript levels of Fos, Fosb, and Fosl2 increase from day 15 to the onset of labor [S1 Table].”**

12.     Figure 5, similar to the labouring stage, the author should draw regulatory regions [enhancers] in the quiescent stage.

**RESPONSE: We have made this change, as suggested [see modified Figure 5].**
